# Supplementary material for: Identification of photoperiod-induced specific miRNAs in the adrenal glands of Sunite sheep (Ovis aries)
Source: Front Vet Sci. 2022 Jul 22;9:888207. doi: 10.3389/fvets.2022.888207 (PMC9354845; doi:10.3389/fvets.2022.888207)
Supplement: Supplementary file 1 [file Data_Sheet_1.ZIP › Supplementary file/miRDeep2 file.zip!/miRDeep2 file/Novel_Structure/SP42A3/Novel_484.pdf]

Provisional ID	: NC_019472.2_21765
Score total	: 2
Score for star read(s)	: -1.3
Score for read counts	: 0
Score for mfe	: 1.7
Score for randfold	: 1.6
Score for cons. seed	:
Total read count	: 154
Mature read count	: 154
Loop read count	: 0
Star read count	: 0

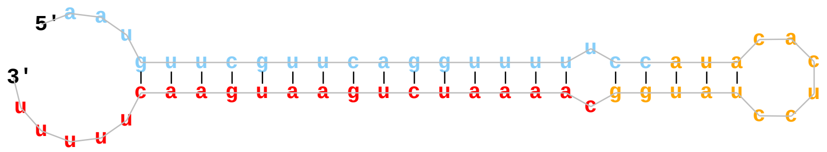

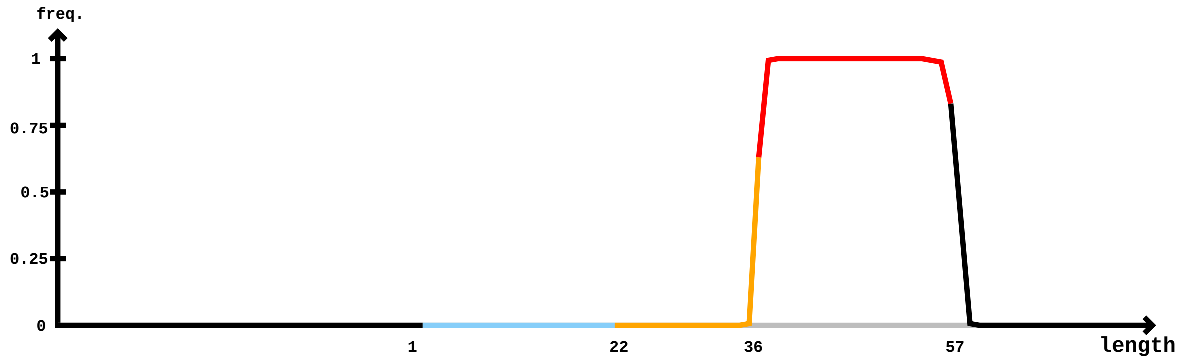


```
                                       Star                               Mature
5'- uugaagcacaaaaagcuuauguauuggguuggccaaaauguucguucagguuuuuccauacacuccuauggcaaaaucugaaugaacuuuuugcccauuugaacucaaucac   -3'    exp
    ...((((.......)))).((.((((((((((.(((((.(((((((((((((((.(((((......))))).))))))))))))))).))))).))).....))))))))).    reads       mm          sample
    ......................................................................gcaaaaucugaaugaacuuuuu....................    1           0           seq
    .......................................................................Aaaaaucugaaugaacuu.......................    1           1           seq
    .......................................................................Gaaaaucugaaugaacuuu......................    1           1           seq
    .......................................................................Aaaaaucugaaugaacuuuu.....................    24          1           seq
    .......................................................................Aaaaaucugaaugaacuuuuu....................    69          1           seq
    .......................................................................Gaaaaucugaaugaacuuuuu....................    1           1           seq
    ........................................................................aaaaucugaaugaaUuuuuu....................    1           1           seq
    ........................................................................aaaaucugaauAaacuuuuu....................    1           1           seq
    ........................................................................aaaaucugaaugGacuuuuu....................    1           1           seq
    ........................................................................aaaCucugaaugaacuuuuu....................    7           1           seq
    ........................................................................aaaaucugaaugaacuuuCu....................    1           1           seq
    ........................................................................aaaaucugaaugaacuuuuuC...................    1           1           seq
    ........................................................................aaaaucugaaugaacuuuuug...................    44          0           seq
    .........................................................................aaaucCgaaugaacuuuuugc..................    1           1           seq
```
